# Supplementary material for: The Genomic Topography of Appendiceal Cancers: Our Current Understanding, Clinical Perspectives, and Future Directions
Source: Cancers (Basel). 2025 Oct 9;17(19):3275. doi: 10.3390/cancers17193275 (PMC12524201; doi:10.3390/cancers17193275)
Supplement: Supplementary file 1 [file cancers-17-03275-s001.zip › Table S1.pdf]

Altered Gene	AC Variant	Percent of Cases per Study (Number of Patients) In Reference Order	Reference(s)
KRAS	NEN	29% (14), 9% (30), 0% (3), 20% (5), 0% (18)	43, 44, 45, 46, 47
GNAS	NEN	0% (14), 3% (30)	43, 44
TP53	NEN	14% (14), 11% (28), 40% (5), 44% (18)	43, 44, 46, 47
SMAD 4	NEN	0% (14), 13% (30), 0% (3), 40% (5)	43, 44, 45, 46
APC	NEN	29% (14), 3% (30), 20% (5)	43, 44, 46
PIK3C A	NEN	0% (14), 0% (37), 0% (3), 0% (5)	43, 44, 45, 46
KRAS	MAA	55% (108), 79% (374), 56% (140), 60% (424), 66% (281), 100% (1), 69% (16), 44% (9), 50% (16), 72% (40), 90% (29), 62% (78), 69% (110), 58% (64), 79% (164), 44% (9), 65% (263)	3, 12, 25, 43, 44, 45, 54, 56, 59, 63, 64, 65, 67, 69, 70, 71, 72
GNAS	MAA	28% (78), 28% (242), 62% (374), 34% (424), 34% (80), 33% (9), 52% (40), 69% (29), 44% (110), 45% (267)	3, 9, 12, 43, 44, 56, 63, 64, 67, 72
TP53	MAA	27% (242), 20% (374), 37% (411), 24% (82), 22% (9), 13% (40), 10% (10), 36% (78), 40% (164), 28% (164), 23% (267)	9, 12, 43, 44, 56, 63, 64, 65, 67, 70, 72
SMAD 4	MAA	17% (242), 23% (320), 18% (426), 15% (86), 3% (40), 10% (10), 18% (78), 20% (44), 11% (164), 8% (267), 54% (13)	9, 12, 43, 44, 63, 64, 65, 67, 70, 72, 103
APC	MAA	9% (242), 4% (374), 12% (427), 7% (88), 0% (10), 15% (78), 12% (242), 16% (44), 2% (164), 7% (267)	9, 12, 43, 44, 64, 65, 67, 70, 72
PIK3C A	MAA	17% (12), 12% (242), 7% (427), 6% (114),13% (40), 16% (78), 7% (44), 7% (163), 5% (267),	9, 12, 43, 44, 63, 65, 67, 70, 72
KRAS	GCA	13% (84), 2% (97), 8% (53), 0% (16), 8% (72), 0% (4), 4% (11), 6% (34), 0% (18), 0% (14) , 0% (16), 6% (34), 0% (18), 0% (14)	12, 43, 45, 47, 70, 72, 75, 91, 93, 94, 97
GNAS	GCA	6% (84), 4 (53), 1% (72), 1% (97), 3% (34)	12, 43, 70, 72, 91
TP53	GCA	33% (84), 28% (72), 14% (97), 20% (11), 6% (34), 6% (18), 0% (16)	12, 70, 72, 75, 91, 93, 97
SMAD 4	GCA	19% (84), 9% (53), 0% (4), 13% (72), 17% (97), 20% (11), 3% (34), 6% (18), 0% (15),	12, 43, 45, 70, 72, 75, 91, 93, 97
APC	GCA	2% (84), 2% (53), 3% (72), 4% (97), 12% (11), 12% (34), 0% (18)	12, 43, 70, 72, 75, 91, 93
PIK3C A	GCA	6% (72), 5% (97), 2% (53), 0% (4), 8% (11), 0% (18)	48, 50, 51, 53, 55, 89
KRAS	CTA	56% (208), 47% (68), 50% (2), 75% (4), 55% (183), 68% (37), 36% (11), 43% (7)	12, 44, 45, 59, 67, 70, 72, 93
GNAS	CTA	25% (208), 17% (23), 31% (183), 14% (37), 18% (11)	12, 44, 67, 70, 72
TP53	CTA	47% (208), 32% (22), 50% (2), 40% (183), 54% (37), 18% (11), 71% (7)	12, 44, 45, 67, 70, 72, 93
SMAD 4	CTA	18% (208), 22% (23), 10% (183), 24% (37), 9% (11), 0% (7), 63% (8)	12, 44, 67, 70, 72, 93, 103

APC	CTA	17% (208), 32% (22), 50% (2), 10% (183), 27% (37), 14% (7)	12, 44, 45, 67, 70, 93
PIK3CA	CTA	3% (29), 6% (183), 27% (37), 29% (7)	44, 67, 70, 93
KRAS	SRC	35% (37), 7% (41), 15% (27), 27% (34)	12, 44, 67, 72
GNAS	SRC	8% (37), 0% (14), 4% (27), 6% (34), 0% (7)	12, 44, 67, 72, 107
TP53	SRC	43% (37), 15% (13), 33% (27), 32% (37)	12, 44, 67, 72
SMAD4	SRC	30% (37), 14% (14), 11% (27), 21% (34)	12, 44, 67, 72
APC	SRC	11% (37), 7% (14), 0% (27)	12, 44, 67
PIK3CA	SRC	0% (14), 0% (27), 3% (34)	44, 67, 72

Table S1. Associated gene mutation frequency data and corresponding references by subtype.
